# Individual‐Based Models Reveal Population Recovery and Impacts of Woodland Creation on Pine Martens ( *Martes martes* ) in Britain

**DOI:** 10.1002/ece3.74175

**Published:** 2026-08-13

**Authors:** Eleanor R. Scopes, Nora Kerecsenyi, Kevin Watts, Jenny MacPherson, Patrick G. R. Wright, Catherine M. McNicol, Jamie Kingscott‐Edmunds, Cally Ham, Matt Guy, Chloe C. Bellamy

**Affiliations:** ^1^ Forest Research Forestry Commission National Office Bristol UK; ^2^ Forest Research Northern Research Station Roslin UK; ^3^ Forest Research Alice Holt Lodge Farnham UK; ^4^ Vincent Wildlife Trust Eastnor, Ledbury UK; ^5^ British Association for Shooting and Conservation Marford Mill Wrexham UK; ^6^ Gloucestershire Wildlife Trust Gloucester UK

**Keywords:** individual‐based models, pine marten, RangeShifter, reintroduction, woodland creation

## Abstract

Individual‐based models (IBMs) predict distribution and abundance patterns resulting from individual life histories. They are useful for simulating spread, particularly in reintroductions, and testing multiple conservation actions. The accuracy of IBMs relies on the parameters used, and recently, calibration methods have been developed to integrate disparate information. We calibrate an IBM for the pine marten ( *Martes martes* ) in Britain using observed presence/absence data. We then use this model to investigate whether the multiple reintroduction projects in Southwest Britain are likely to coalesce into a single population, and whether woodland creation can be designed to facilitate dispersal. Our calibrated model reveals that some populations in Southwest Britain may remain isolated after 150 years, likely due to a lack of residential habitat between population centres under our definition of suitable habitat. However, they remain successful at a local scale, with populations persisting in all release zones. We find that none of our woodland creation scenarios, which vary in the area and configuration of new patches, increase the abundance of pine martens or their dispersal capacity. Instead, the biggest factor influencing outcomes is the landscape. Our results suggest that realistic tree planting, in particular patch size, is insufficient to benefit pine martens under our model definitions. However, the coarse scale of our model and our definition of suitable patches could underestimate the influence of small woodlands and hedges on population dispersal and viability. We show that the reintroduction of pine martens to England and Wales successfully restores parts of the former range into the near future. Woodland cover is an important factor in landscape carrying capacity, but significant woodland creation may be needed to enact meaningful change. Our calibrated model can support the selection of reintroduction sites with the greatest probability of facilitating population coalescence.

## Graphical abstract

We used a calibrated individual‐based model to explore the reintroduction of pine martens to Britain and whether woodland creation can be targeted to improve pine marten dispersal. We find that the reintroduction programme is successful into the near future, even if it does not appear to form a single unified population. None of the woodland creation scenarios we tested improved pine marten abundance or dispersal, though our model may underestimate the impact of small woods and hedges on chosen paths.

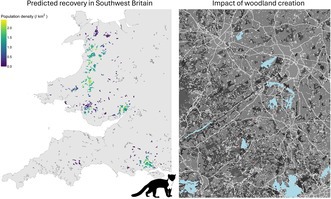

## 1 Introduction

Reintroductions are an important tool to restore species to their former range where they cannot easily return (Ovenden et al. 2019), or to adapt to shifts in suitable habitat due to climate change (Oliver et al. 2012). However, reintroduction projects are also high cost and high‐risk endeavours (Hunter‐Ayad et al. 2020), and it can be difficult to predict the behaviour of released individuals (McNicol et al. 2020). This means reintroduction projects require careful planning and monitoring to assess their feasibility and success.

Pine martens ( *Martes martes* ) are a medium‐sized mustelid, native to Europe (MacPherson et al. 2024). In Britain, the pine marten population underwent a severe reduction and restriction in the early 19th century and, although it has been increasing in Scotland, recovery has been minimal in England and Wales (Sainsbury et al. 2019). There is particular interest in restoring pine marten due to its protected species status (e.g., Schedule 5, Wildlife and Countryside Act 1981), and their possible role in controlling invasive grey squirrel ( *Sciurus carolinensis* ) populations, as has been reported in Ireland (Sheehy and Lawton 2014). To aid the restoration of a viable pine marten population in Southern Britain, a reintroduction project was proposed (MacPherson and Wright 2021; MacPherson et al. 2024). Individuals were translocated from Scotland to Wales in 2015–2017, and to the Forest of Dean in 2019–2021, following feasibility studies (MacPherson et al. 2014; Stringer et al. 2018). A population of pine martens is also present in the New Forest and, although the origins are unclear, sightings have been reported since 1993 (Ward 2021). Further reintroductions were also proposed in Devon and Somerset (MacPherson et al. 2024) and took place in 2024 and 2025, respectively (Hamston et al. 2023), which created five pine marten populations in Southwest Britain. Although each reintroduction is designed to be self‐sustaining, it is valuable to consider whether these populations will coalesce into a larger, more resilient population and continue to spread further east. Understanding the spread of these populations will help conservation bodies plan monitoring and public communication efforts. Pine marten presence also impacts the types of lethal control that can be used on grey squirrels, which has repercussions for government grants for grey squirrel management and control within their expanding range (Gill et al. 2019).

The existing pine marten reintroduction sites were chosen based on habitat suitability models, which identified woodland as the most important feature (MacPherson et al. 2014, 2024; MacPherson and Wright 2021; Scopes et al. 2025). However, this reliance on woodland could mean that pine martens have difficulty dispersing and establishing in other areas. Woodland covers only 14% of the UK's land area and 10% of England's, although total tree canopy cover is estimated at 14.9% for England (Braby 2024). Large areas of Central and Eastern England have low habitat suitability for martens due to the patchy coverage of small woodlands (MacPherson et al. 2024; Scopes et al. 2025). Multiple tree planting targets have been set across the devolved governments to improve biodiversity and meet the UK's net zero commitments (Ares et al. 2021). It is estimated that meeting these woodland creation targets could increase the pine marten population by 31% if individuals are able to colonise all new woodland areas (Hunter et al. 2026). There is therefore an opportunity to direct woodland planting to increase habitat suitability and dispersal potential of landscapes for pine marten, in reintroduction areas or where populations are naturally recovering.

Individual‐based models (IBMs), which simulate interactions between individuals to generate metapopulation patterns in abundance and distribution, are critical tools in planning conservation actions (DeAngelis and Mooij 2005). For reintroductions, IBMs can integrate information on habitat, population demographics and dispersal to effectively model the spread from release sites and thus predict population persistence (Hunter‐Ayad et al. 2020).

RangeShifter is an example of an individual‐based model which can simulate demography, dispersal and genetics in a flexible, stochastic and spatially explicit manner (Bocedi et al. 2021). Although it is restricted to modelling a single species, rather than species interactions, it can simulate heterogeneous dynamic landscapes and complex species life histories. One limitation of RangeShifter is that during reproduction, juveniles are assigned to the same patch as the mother, but not necessarily the same cell, meaning juveniles can start dispersal on the other side of a patch (Ovenden et al. 2019). The model also only allows individuals to disperse once in their lifetimes, which might underestimate connectivity resulting from changes in home range (Almela et al. 2021). RangeShifter has been used to explore potential reintroduction sites (Ovenden et al. 2019), compare control measures for invasive species (Almela et al. 2021), and to prioritise actions to combat climate change and habitat fragmentation for multiple species and diverse landscapes (Synes et al. 2020).

IBMs can require considerable data to function effectively and require the user to input accurate parameters (e.g., survival) to model populations robustly (Hunter‐Ayad et al. 2020). Parameters can be drawn directly from existing literature (Ovenden et al. 2019) or expert opinion. Recently, a new method has been developed to integrate all data sources through Bayesian inference (Malchow et al. 2024). In this method, parameters from the literature and expert opinion, alongside their uncertainty, can be used to generate a prior. Then, the prior can be updated through Bayesian comparison to observed data to generate an updated posterior distribution (Malchow et al. 2024). This method has the benefit of integrating multiple data sources to make the best use of available information for species of conservation concern which may not be well studied. Bayesian calibration also allows for uncertainty to be propagated from data sources to model projections (Malchow et al. 2024), which is beneficial for understanding potential limits of the model. Calibration can therefore allow the creation of more robust IBMs, which can support sound conservation decision‐making.

In this study, we aim to calibrate a RangeShifter model using literature, expert opinion and observed pine marten presences. We then employ the calibrated model to (1) explore the potential spread of pine martens from the existing and proposed reintroductions in Southwest Britain, and (2) investigate whether realistic woodland creation scenarios have the potential to enhance pine marten abundance and distribution.

## 2 Methods

### 2.1 RangeShifter Model

All data analyses were conducted in R version 4.4.2 (R Core Team 2024). We used the package *RangeshiftR* version 1.1.0 (Malchow et al. 2021) to create a model of pine marten life history. The model simulates yearly timesteps, with reproduction, movement and survival occurring in order each year.

Our landscape is based on the Corine 2018 land cover data (Büttner et al. 2004) at 100 m resolution, simplified into 12 habitat groups (Table S1), with added information on roads and trees outside of woodland (TOW). We added the locations of motorway, major and minor (A‐ and B‐roads) roads from the OS Open Roads database (version 2.4, OS data Crown copyright 2023; https://www.ordnancesurvey.co.uk/products/os‐open‐roads), with roads overwriting all overlapping habitats, including TOW. TOW comprise hedges, tree lines, individual trees and small woodland patches (under 20 ha). We used TOW data from Liu et al. (2023), which gives canopy height at the 30 m resolution. We converted this to TOW presence and re‐scaled to 100 m resolution, retaining cells with 75% coverage of TOW. When adding this data to the landscape, it took priority over all habitats apart from woodland and urban habitats. The original TOW data can overestimate TOW in urban areas due to the density of buildings (Liu et al. 2023), hence the prioritisation of urban habitat.

We ran a patch‐based RangeShifter model (full specification Table S2), with simple reproduction (sexual reproduction but without the formation of a harem). Patches were defined as contiguous areas of woodland at least 2 km^2^ in size, separated by at least 100 m of another habitat (Figure S1). This patch composition represents the main component of pine marten habitat suitability (MacPherson and Wright 2021; Scopes et al. 2025) and the minimum area of good habitat required to support an adult pine marten in temperate woodland (Zalewski and Jedrzejewski 2006; MacPherson and Wright 2021). To account for pine marten foraging behaviour outside woodland (Caryl et al. 2012), we added a 100 m buffer around each woodland patch and allowed habitats used by pine martens (scrub, heath, natural grasslands and TOW) to be included in the patch area.

We implemented a model with three life stages: juvenile, sub‐adult and adult. Survival between stages occurred each year, such that individuals were juveniles in their first year, sub‐adults in their second and adults in years three and onwards. Sub‐adults were the only class to disperse (Larroque et al. 2015), and adults were the only class to reproduce. We implemented a negative effect of adult and sub‐adult density on survival (Zalewski and Jedrzejewski 2006), using the same survival value for all habitats in a patch.

To model pine marten dispersal, we used a stochastic movement simulator (SMS). We calibrated directional persistence, the degree of correlation between movements, as the SMS is most sensitive to this variable (Coulon et al. 2015). We used a perceptual range of 500 m, a memory size of the 5 pixels, no goal type and a harmonic mean method, reflecting similar RangeShifter models on carnivores (Ovenden et al. 2019). Each habitat was given a cost of movement for the SMS and a per step mortality risk, based on the relative ease and risk of movement compared to woodland (Table S3). Costs and mortality risks were chosen based on a similar model for British carnivore (Ovenden et al. 2019), altered to reflect the more arboreal movement of pine martens, and reviewed by the pine marten experts amongst our authorship team (J.M., P.G.R.W., C.M.M.).

Dispersing individuals could move a maximum of 70 km in a year (Scopes et al. 2025), up to a maximum of 500 km in their lifetimes. We modelled a sigmoid density‐dependent effect on settlement similarly to Malchow et al. (2024): we fixed the maximum probability of settlement at one and the slope as −5, and only calibrated beta (the inflexion of the slope). Calibrating this single parameter at these fixed values allows the exploration of a wide variety of density‐dependent relationships (Malchow et al. 2024).

We based initial populations on the actual reintroduction locations and years. In Wales, 20 individuals were released in 2015, followed by a further 19 and 12 individuals in 2016 and 2017, respectively. In the Forest of Dean, 18 individuals were released in 2019 and 17 in 2021. As the extent and size of the New Forest population in 2015 are unclear, we initiated the model with pine martens in only two patches of woodland where we had direct evidence of presence in 2015. This is a conservative estimate of where pine martens occurred in the New Forest at the time, as monitoring was restricted to a few locations. Initialising in only two patches could prevent the population from establishing in the model, which would disagree with the observed data. We therefore initialised the patches with a relatively high density of pine martens (one per km^2^) to ensure the population would expand appropriately.

### 2.2 Calibrating the RangeShifter Model

#### 2.2.1 Pine Marten Calibration Data

**FIGURE 1:**
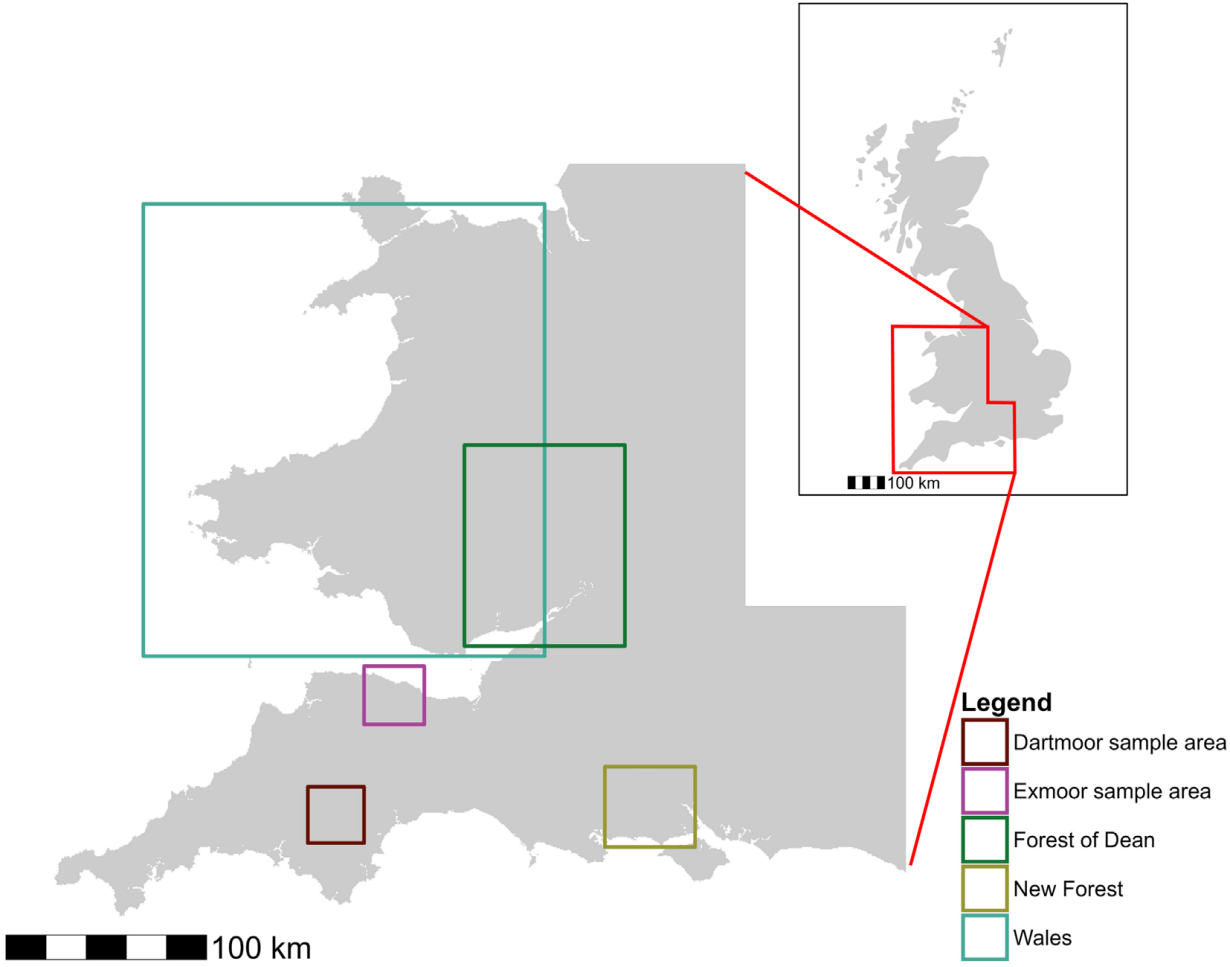
Map of the study area in Southwest Britain. The inset map shows Great Britain and indicates in red the study area. The main map shows in grey the landscape extent of the model used for calibration and spread in the Southwest. The areas of observed data collection are shown: Wales (teal), the Forest of Dean (dark green) and the New Forest (olive green). The sample area for Dartmoor in Devon (dark red) and Exmoor in Somerset (pink) release sites are also shown.

We calibrated the RangeShifter model for pine martens against observed data from three populations in Southwest Britain: Wales, the Forest of Dean and the New Forest (Figure 1). For the pine marten reintroductions to Wales and the Forest of Dean, monitoring included radio‐collar telemetry, opportunistic and structured camera trap surveys, scat transect surveys and verified sightings from the public. Telemetry data were gathered up to 15 months post‐release from the initially translocated martens (McNicol et al. 2020), and consisted of 2055 telemetry fixes in Wales and 1244 fixes in the Forest of Dean.

Scat transects were conducted in 2019 in Wales and 2020–2023 in the Forest of Dean to monitor population expansion. We only included scats which had been verified as pine marten through DNA testing, as relying on morphology alone can result in false positives for pine martens (Davison et al. 2002). Scats from the Welsh survey were analysed at Waterford Institute of Technology, Ireland. To isolate DNA from scat samples approximately 0.2 mg of scat material was transferred to 1 mL S.T.A.R buffer (Stool Transport and Recovery buffer, Roche), vortexed and allowed to stand for at room temperature for > 30 min. The samples were centrifuged at 1000 *g* for 1 min and then DNA was extracted from 150 μL of the supernatant using the ZR Genomic DNA II Kit (ZYMO Research, California, USA). Pine marten and fox were identified using the qPCR TaqMan species specific assays as described in O'Reilly et al. (2008) for fox and Mullins et al. (2010) for pine marten. The Welsh survey yielded 137 scats, of which only three tested positive for pine marten DNA, and a further 21 transects where no scats were found. None of the scats found in the Forest of Dean underwent DNA testing, and so this data was not used in our analysis.

Public sightings were submitted from 2019 to 2023 for the Forest of Dean project, and 2018 to 2019 for the Welsh project, and we only included public sightings in our analysis that had been verified by an expert. This resulted in 48 verified sightings in Wales and seven in the Forest of Dean. Finally, camera surveys were conducted in 2022 and 2023 for the Forest of Dean, producing nine presences, and 2017–2020 in Wales, providing 99 presences. Unfortunately, there were no records of where camera traps were placed but returned zero records of pine marten presence, so the negative scat transects provided the only absence data.

Data from the New Forest consisted of verified public sightings and structured camera trap surveys in 2021 to 2023. Although observations exist from 1993 (Ward 2021), we used data from 2015 onwards to match the dates of the first reintroduction to Wales. In the New Forest, there were 111 opportunistic sightings and camera trap presences and 186 presences from the structured surveys.

We thinned the observed data to the patch‐level (detailed below), prioritising presences over absences where they occurred in the same patch in the same year. This is because absences only occurred from negative scat transects on forest roads and so were likely to miss individuals which had been detected through other methods. Once thinned, the data provided 89 habitat patches with presence/absence data from 2015 until 2023.

#### 2.2.2 Calibration

We calibrated the RangeShifter model using a method for Bayesian inference created by Malchow et al. (2024). We altered this method to calibrate against binary presence/absence data instead of abundance data. Following Malchow et al. (2024), in each iteration, the population response is simulated ten times and the outputs averaged to estimate mean occupancy for each patch, each year. We then altered the method by using a binomial distribution to compare the mean occupancies in each iteration to the observed data. We refer to the binomial parameter as ‘detection probability’ and calibrate it within the model. We calibrated eight model parameters: survival of juveniles, sub‐adults and adults, fecundity, the density‐dependent effect on survival, emigration probability, settlement slope beta and directional persistence.

We created priors for each parameter based on a truncated normal distribution. We selected prior values from the literature and test models (Table 1). We used test models to select priors for survival density‐dependence and settlement slope beta as we could not find analogous variables in the literature. The prior for survival density‐dependence was created based on applying the model to a simplified landscape of entirely suitable habitat (a woodland patch) and reviewing the density of individuals in the patch at population equilibrium. The mean survival density dependence (0.04; Table 1) gives a density of one per km^2^, with the range of variables restricting density to a maximum of 2.5 per km^2^, which is within the range for densities of pine marten in Europe (Zalewski and Jedrzejewski 2006; Sheehy and Lawton 2014). The settlement slope beta prior was created using the *plotProbs* function from *RangeShiftR* package to form a variety of different shapes for the relationship between density and settlement probability.

**TABLE 1:** The prior distributions for all parameters calibrated in the RangeShifter model.

Parameter	Mean	SD	Minimum	Maximum	References
Adult survival	0.880	0.200	0.010	0.990	Powell et al. (2012)
Sub‐adult survival	0.600	0.200	0.010	0.990	Ruette et al. (2015)
Juvenile survival	0.500	0.300	0.010	0.990	Ruette et al. (2015)
Fecundity	2.740	1.350	1.000	5.000	Kleef and Wijsman (2015)
Survival density‐dependence	0.040	0.100	0.005	0.100	
Sub‐adult emigration	0.540	0.200	0.010	0.990	Larroque et al. (2015)
Settlement slope Beta	1.500	1.000	0.100	5.000	
Directional persistence	5.000	2.000	1.000	10.000	Ovenden et al. (2019)
Detection probability	0.500	0.300	0.010	0.990	

*Note:* The priors are normal distributions based on the mean and standard deviation (SD) and truncated between the minimum and maximum values. Means and standard deviations were drawn from the given references, or from test models as detailed above if blank. We specified a vague prior for detection probability.

The model was run for the full length of the observed data: from 2015 until 2023. We ran the model with three chains for 5000 iterations, with 600 iterations discarded as burn‐in, which produced adequate convergence as indicated by Gelman‐Rubin statistics < 1.1 for each parameter (Gelman and Rubin 1992). The Gelman‐Rubin statistic describes the convergence of the simulations by comparing the variance within and between the three chains, with a value close to one indicating that the simulations have converged.

The method created by Malchow et al. (2024) includes spatial fold cross‐validation, which limits potential inflation of accuracy measures caused by any spatial autocorrelation in the input data (Veloz 2009). We split the observed data into four folds based on latitude, with each fold containing a similar number of patches. One fold contained all of the data from the New Forest. For the cross‐validation, each spatial fold in turn was left out of the calibration process. The predicted occupancy probability from the calibrated model was then compared to the independent data left out of the model using area under the curve (AUC), which indicates disagreement (0), random fit (0.5) or agreement (1; Shabani et al. 2018). We could not calculate AUC for the New Forest spatial fold as this data contained only presences. We therefore also calculated the sensitivity of the calibrated model, using a presence/absence output, to the independent data. To create the binary output, we created a threshold to assign occupancy probability to presence or absence categories using the ‘p10’ method. The ‘p10’ method identifies the occupancy probability for all of the presences used in the model calibration and sets the threshold for partition so that the bottom 10% of probabilities fall into the absence category (Pearson et al. 2007). We present the AUC and sensitivity of the model to the spatial folds, but use all of the data to estimate the posterior values.

### 2.3 Spread in the Southwest

We used the same RangeShifter model described above to estimate marten spread in the Southwest, with the mean of the posterior for all calibrated parameters (Table S2; Table 2). We ran this model with 100 replicates for 150 years, matching the time for woodland maturation in the analysis below. The model included the same initial populations (reintroductions to Wales and the Forest of Dean, existing population in the New Forest) but also included the recent reintroduction to Devon and Somerset, releasing individuals in Dartmoor (Devon) in 2024 and Exmoor (Devon and Somerset) in 2025. To replicate this, we defined areas of release in Dartmoor and Exmoor (Figure 1) and randomly selected three patches in each location to act as release sites which remained the same between replicates. We then randomly assigned twenty individuals, with even sex ratio, to the patches for each release (2024 and 2025). The model produced SMS heatmaps showing the number of visits dispersing individuals make to each pixel over the 150 years. We used this data to present the proportion of visits which occur in each habitat type, alongside the proportional availability of each habitat in the landscape. We removed ‘Salt water’ from this analysis as it is not used by pine martens.

**TABLE 2:** The posterior estimates for each parameter calibrated in the RangeShifter model.

Parameter	Mean	LCI	UCI
Density dependence	0.051	0.007	0.097
Fecundity	3.387	1.729	4.807
Juvenile survival	0.688	0.307	0.958
Sub‐adult survival	0.679	0.369	0.959
Adult survival	0.756	0.469	0.964
Emigration probability	0.713	0.409	0.962
Settlement parameter	1.505	0.181	3.162
Directional persistence	5.312	2.452	8.613
Detection probability	0.237	0.109	0.449

*Note:* The mean, lower confidence interval (LCI) and upper confidence interval (UCI) are displayed.

### 2.4 Woodland Creation Scenarios

We based the woodland creation scenario analysis on Synes et al. (2020), who explored the success of six different conservation actions for eight ‘virtual’ species in six in landscapes with different levels of habitat fragmentation. We completed a factorial experiment combining six landscapes, four woodland creation methods, two levels of woodland creation and two types of initial populations (e.g., Figure S1). For each of the 96 combinations of scenarios, we created ten replicates which randomly placed woodland within the landscape according to the given method and level (Synes et al. 2020). For each combination, we also ran 100 population replicates, as RangeShifter is stochastic, and considered the average across population replicates in the results.

We compared the scenario outputs to a baseline model with no habitat changes, creating a different baseline for each landscape and initial population scenario. We present mean population abundances over 150 years for each scenario combination in comparison to this baseline.

#### 2.4.1 Selecting Landscapes

We randomly sampled six 40 km by 50 km landscapes from Britain with different proportions of woodland cover, aiming for evenly spaced gradations from 5% to 25% cover and increasing proportions of woodland from landscape one to six. We selected this landscape size to limit edge effects from wide ranging individuals. We rejected landscapes with less than three suitable patches for pine martens (as defined above) to allow some population dispersal.

#### 2.4.2 Defining Woodland Creation Methods

We considered four woodland creation methods: creating random patches of woodland, creating random patches of woodland adjacent (within 500 m) to existing woodland, expanding existing woodland and expanding only small (≤ 25 ha) existing woodlands. For each method, ten patches of woodland were randomly created in available locations, which were identified using ‘woodland opportunity maps’. For England, we used a binary opportunity map created by the Friends of the Earth and Terra Sulis (Terra Sulis Research 2020). For Wales we used the Welsh government woodland creation score map (Open Government Licence v3.0; https://datamap.gov.wales/maps/woodland‐opportunity‐map‐2021/), which we converted to binary data using a threshold of 15 (i.e., the top 60% of scores became suitable for woodland creation). For Scotland, we could not access a current ‘woodland opportunity map’, so we allowed woodland creation in existing areas of scrub, pasture, heath, natural grassland, arable, bare land and TOW. We simulated woodland growth by using dynamic landscapes in RangeShifter, where newly ‘planted’ woodlands progress from scrub to TOW to mature woodland. The patches were redrawn for each additional habitat type, allowing new woodlands to become part of existing patches. We set the transition times as 30 years to transition to scrub, a further 30 years from scrub to TOW and another 20 years from TOW to woodland, according to data from Fuentes‐Montemayor et al. (2021).

#### 2.4.3 Defining Levels of Woodland Creation

The areas of the created woodland were randomly drawn from two levels of effort based on the areas of woodlands planted under the England Woodland Creation Offer (EWCO) from June 2021 to July 2024. This data contained 1346 woodlands. At level 1, the areas of simulated woodland creation were drawn from the smallest 50% of real areas planted under EWCO, which had a mean area of 2.11 ha (standard deviation: 0.98 ha, range: 0.60–4.17 ha). At level 2, the simulated areas were drawn from the largest 50% of EWCO sites, which had a mean area of 16.17 ha (SD: 18.25 ha, range: 4.19–216.64 ha).

#### 2.4.4 Defining Initial Populations

We tested two types of initial pine marten population: established and reintroduced. We simulated a putative reintroduction (not based on historical locations) by initiating the model with 40 individuals, with equal sex ratio and equal numbers released in the first two years, following the numbers of individuals reintroduced to Wales (McNicol et al. 2020). We randomly selected a single patch and its two closest neighbouring patches as the starting location for putative reintroduction. Each individual was randomly assigned one of these three patches.

For the established population, we initiated the model with a density of 0.25 individuals per km^2^ for all patches, rounded to the nearest whole number. This density matches the established populations in Scotland (Caryl et al. 2012; Croose et al. 2019). The same initial populations were used for each landscape, regardless of the woodland creation scenario and effort applied.

## 3 Results

### 3.1 Model Calibration

#### 3.1.1 Posterior Estimation and Cross‐Validation

**FIGURE 2:**
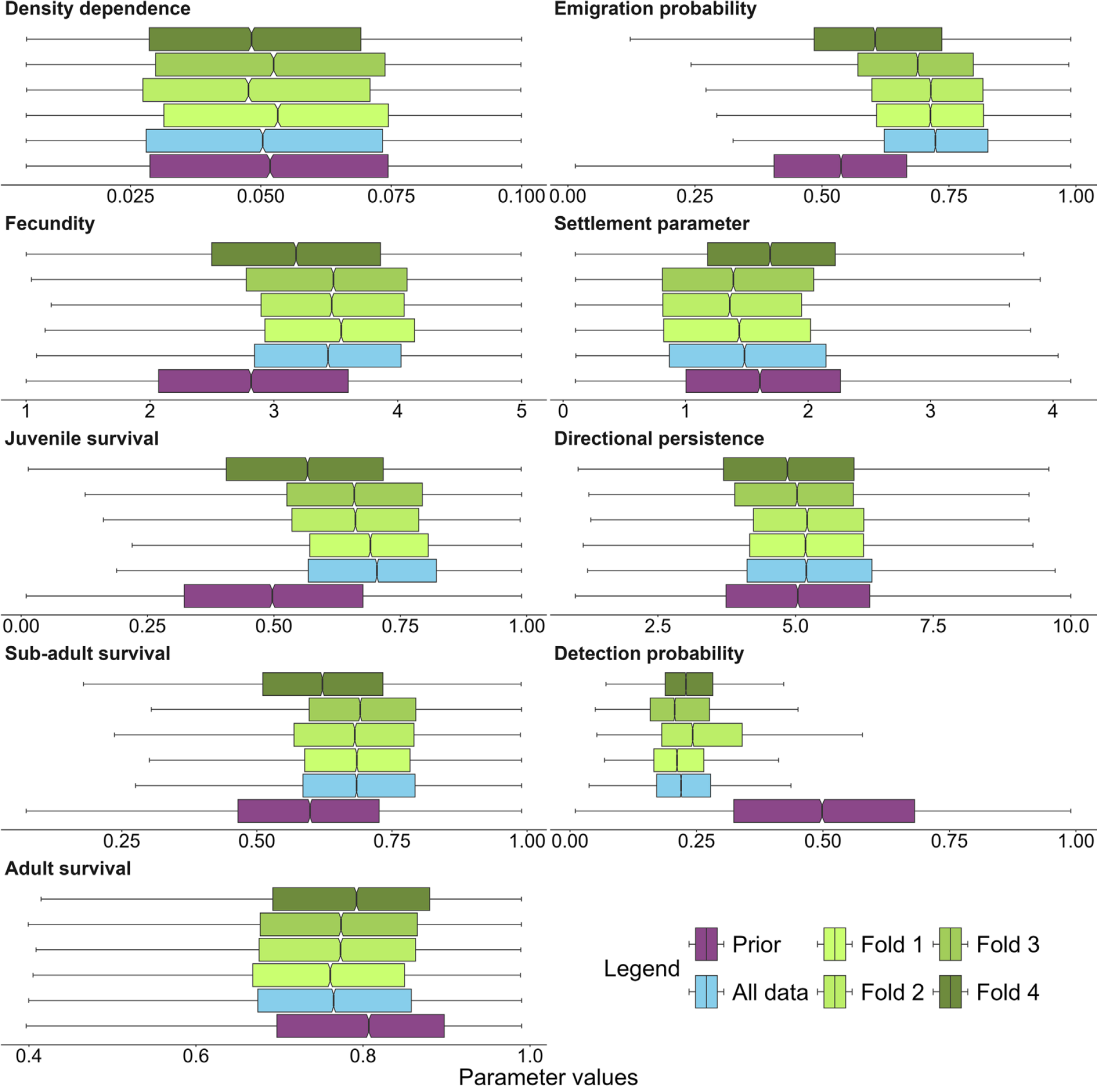
The prior distribution (purple) and the posterior distribution for the calibrated models run with all of the data (blue) and without each spatial fold (greens). The box plot indicates the mean, lower and upper quantiles, and range of the corresponding distributions. Note the *x*‐axis scale changes for each parameter, depending on the specified prior.

The Bayesian calibration of the RangeShifter model provides posterior distributions for eight model parameters we chose to calibrate, and the detection probability linking the model to the observed data. The posterior distributions for the entire observed dataset, and each spatial fold, are shown in Figure 2, with comparisons to the prior distribution we selected for each parameter. We expected that the posterior distributions would show some difference to the relevant prior distributions, as the priors are solely based on the literature and posteriors incorporate additional information from comparisons to the observed data. These differences would be reflected in Figure 2 as a narrowing of the range and quantiles, and a shift in the mean, as can be seen for sub‐adult survival. We found that detection and emigration probabilities showed the greatest change from prior to posterior distribution (Figure 2), with emigration probability posterior increasing on average compared to the prior and detection probability decreasing. However, some of the parameters show minimal changes from the prior to posterior, for example density dependence, suggesting that there was insufficient information in the observed data to influence all parameters. Table 2 shows the posterior means used as parameter values in the rest of the analysis.

The results of the spatial fold cross validation are shown in Table 3. Overall, the model using all of the data has a moderate mean AUC value, representing modest fit with the observed data. The mean AUC for all spatial folds is > 0.5, suggesting better than random fit, though it is marginal for fold 1. This poor fit of fold 1 is also reflected in the sensitivity scores, which suggest only 18.5% of presences in fold 1 were correctly attributed by the model. Fold 2 and 3 show moderate sensitivity, whilst fold 4 shows the greatest sensitivity, with 90.9% of presences correctly attributed (Table 3).

**TABLE 3:** The area under the curve (AUC) and sensitivity of the model run with all data and without each spatial fold.

Fold	AUC			Sensitivity
	LCI	Mean	UCI	
All data	0.684	0.753	0.822	
Fold 1	0.428	0.568	0.708	0.185
Fold 2	0.495	0.66	0.825	0.522
Fold 3	0.715	0.811	0.906	0.558
Fold 4				0.909

*Note:* For AUC, the mean, lower confidence interval (LCI) and upper confidence interval (UCI) are displayed. We could not calculate AUC for fold four as it only contained presences. We could not calculate sensitivity for all of the data as the comparison is circular: the pine marten observation data are used to create the threshold of the binary output, which would then be compared to the same observation data.

### 3.2 Spread in Southwest Britain

**FIGURE 3:**
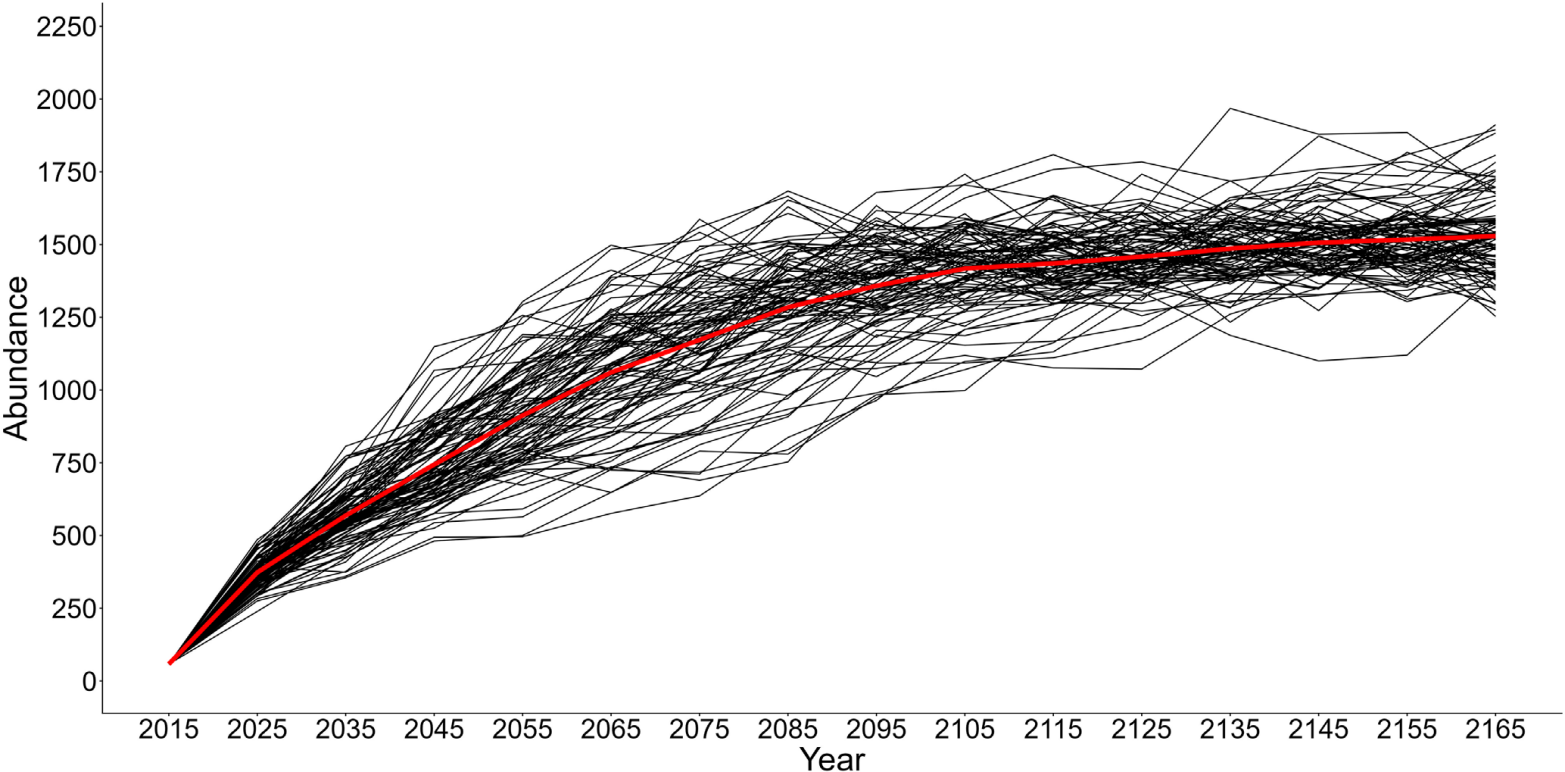
The abundance of pine martens in the Southwest of Britain over 150 years based on simulated populations from a calibrated RangeShifter model. Each of the 100 replicates is shown (black), alongside the mean abundance across replicates (red).

**FIGURE 4:**
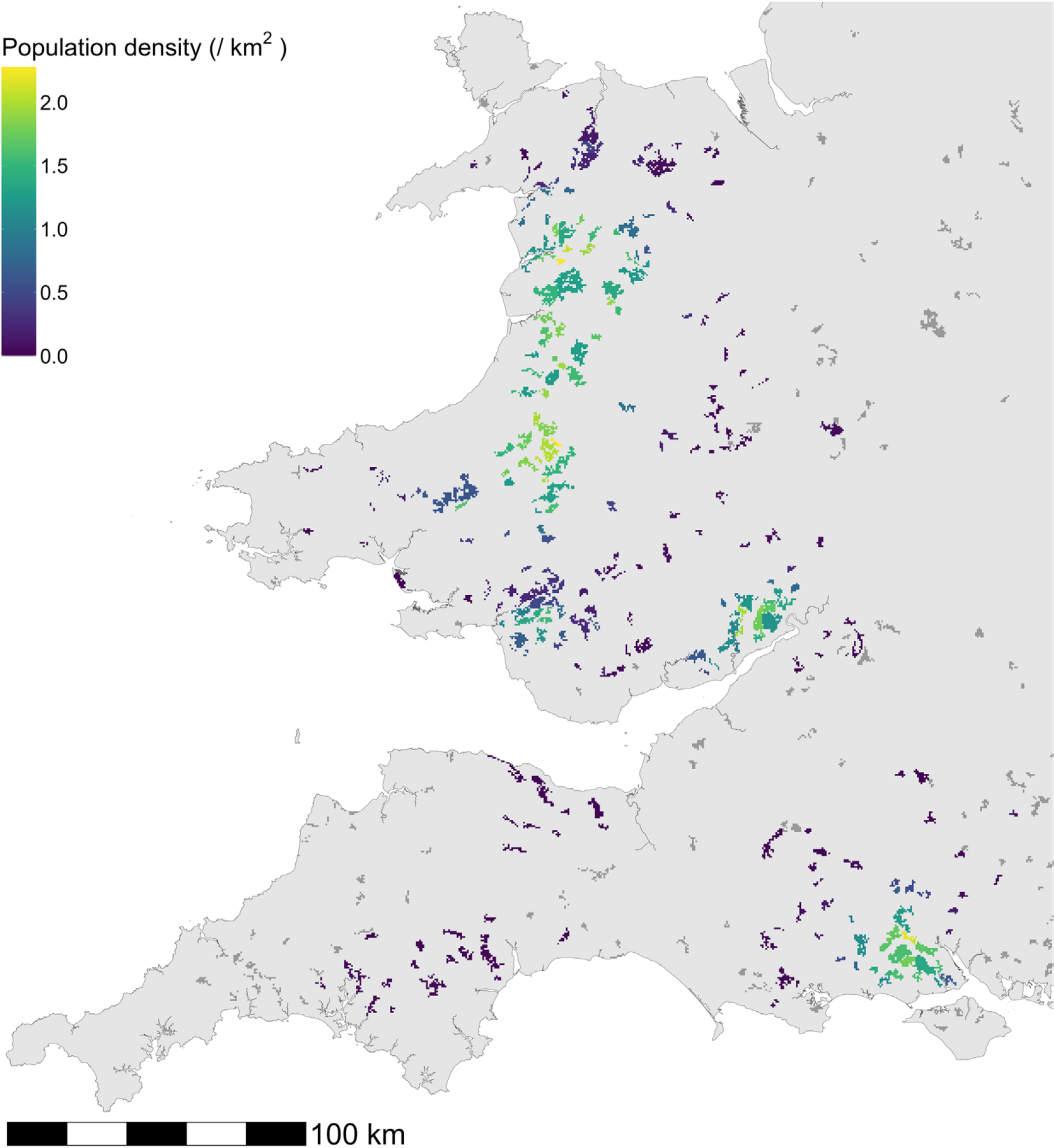
The mean population density (per km^2^) in year 150 in the Southwest of Britain. The colour gradient shows the mean predicted population density in each habitat patch in the year 150 across 100 model replicates. Dark grey patches indicate available suitable habitat patches that are unoccupied.

The calibrated model, using the posterior means for the relevant parameters (Table 2), shows population growth of pine martens in the Southwest which begins to plateau around year 100 (i.e., 2115) at around 1500 individuals (Figure 3). The population density for all patches in year 150 shows extensive occupancy in Wales, the Forest of Dean and the area surrounding the New Forest (Figure 4). However, there is low density in Devon and Somerset, suggesting the reintroduction here may not be successful under our model conditions.

**FIGURE 5:**
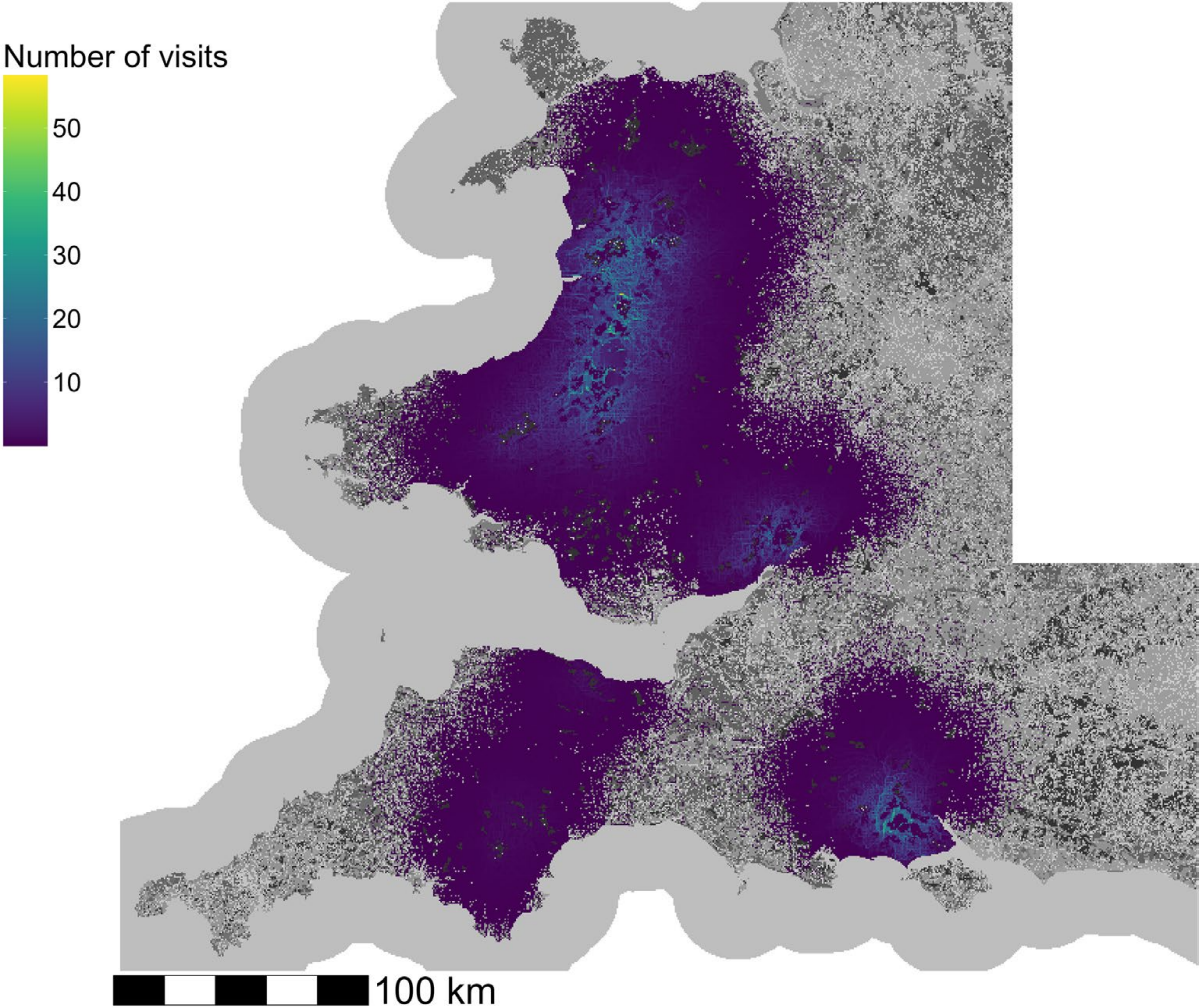
A heatmap of number of visits to each pixel of all dispersing individuals over 150 years in the model of southwest Britain. The colour shows the number of visits to each pixel on a grey‐scale landscape map of the different habitat types. The map shows mixing between populations.

**FIGURE 6:**
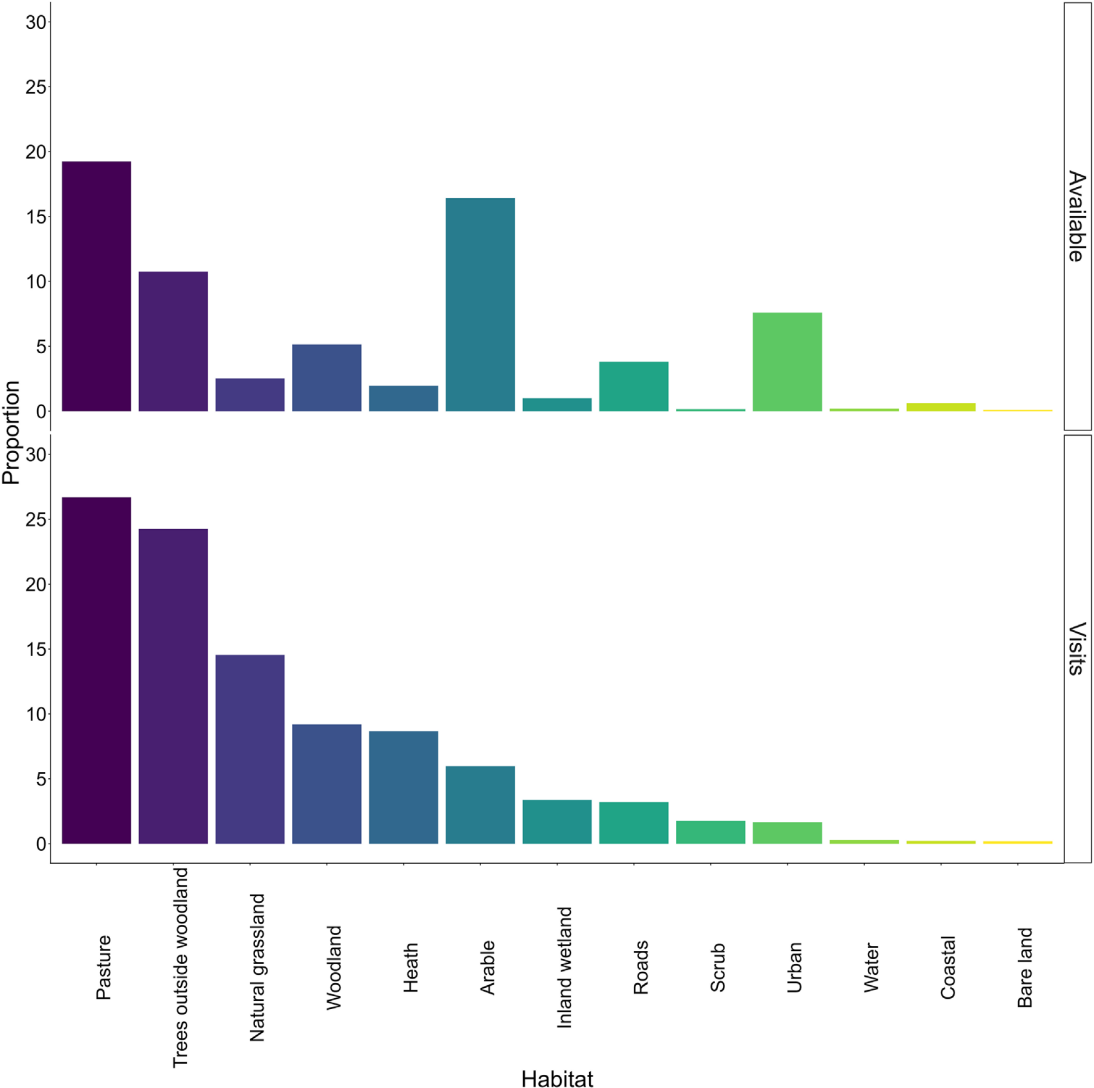
The proportion of each habitat available in the landscape (top panel) and the proportion of visits made to each habitat by dispersing individuals (bottom panel) in the model of Southwest Britain. The proportion of visits is out of all the steps taken by dispersing individuals across the 150‐year run time. The habitats are ordered by proportion of visits. Salt water has been removed as it is not used by pine martens.

The SMS heatmap (Figure 5) shows the number of visits by dispersing individuals across the 150 simulation and suggests some connectivity, through individual movement, between the population centres. There appears to be moderate connectivity between Wales and the Forest of Dean, and limited connectivity between the reintroduced populations in Devon and Somerset. However, the heatmap also suggests that there is no movement of individuals connecting the New Forest to the other populations, nor connecting the Devion and Somerset populations to those further east. Habitat use by dispersing individuals is not always the same as the availability of habitat (Figure 6). For example, pasture and arable habitats have similar availability within the tested landscape (15%–20%; Figure 6). However, pasture is used more frequently than expected by dispersing individuals (~26%; Figure 6), and arable is used less frequently (~5%; Figure 6). This is directed partly by the habitat‐specific costs for dispersal (Table S3), with the model predicting greater proportional use of pasture and TOW than is available.

### 3.3 Woodland Creation Scenarios

**FIGURE 7:**
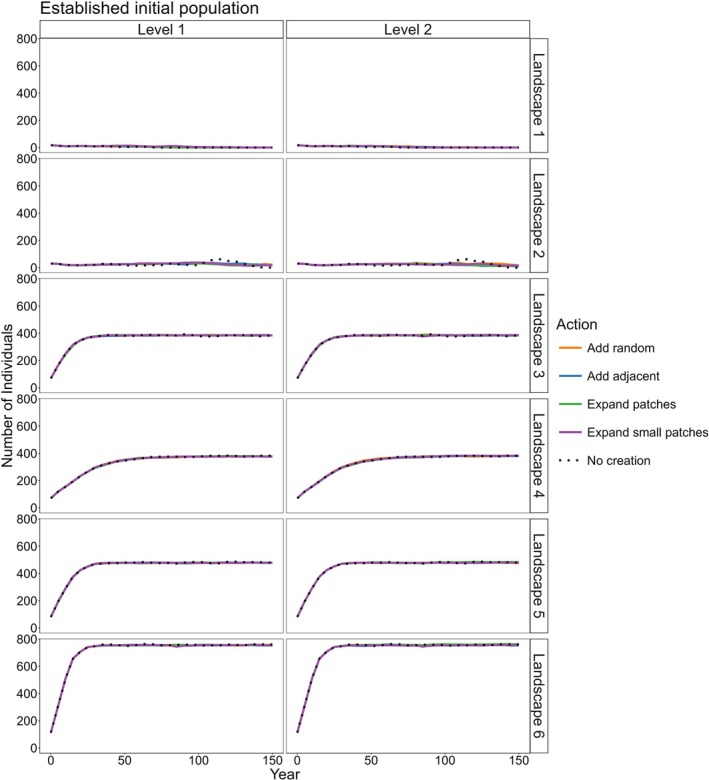
The mean number of individuals over 150 years for each landscape (rows), woodland creation scenario (line colours) and level of creation (columns) compared to no intervention (dotted line) for an initial established population. Level refers to the amount of woodland creation, with level 2 indicating larger creation areas. All populations become extinct in landscapes 1 and 2 (7.6% and 9.8% woodland cover, respectively) by 150 years.

**FIGURE 8:**
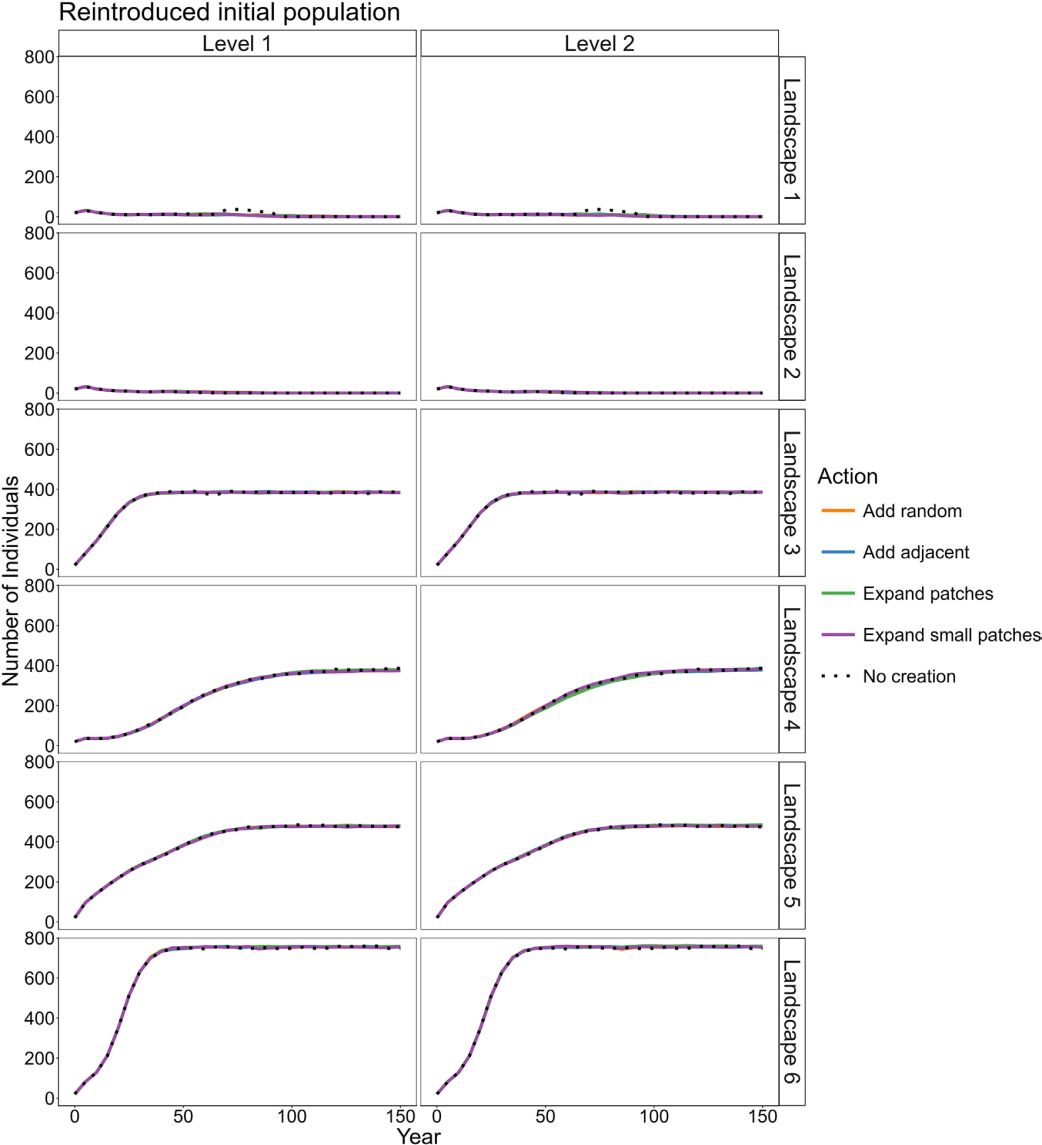
The mean number of individuals over 150 years for each landscape (rows), woodland creation scenario (line colours) and level of creation (columns) compared to no intervention (dotted line) for an initial reintroduced population. Level refers to the amount of woodland creation, with level 2 indicating larger creation areas. All populations become extinct in landscapes 1 and 2 (7.6% and 9.8% woodland cover, respectively) by 150 years.

We found that all woodland creation scenarios had minimal effect on the abundance of the pine marten population over 150 years, whether starting with an established (Figure 7) or reintroduced (Figure 8) population. Landscape, and therefore the proportion of woodland to some extent, had the greatest impact on abundance, with simulated populations becoming extinct in landscapes 1 and 2, and population carrying capacity increasing for landscape 6 (Figures 7 and 8). Reintroduced populations are slower to reach landscape carrying capacity than established populations which are initiated in all patches.

## 4 Discussion

We calibrated a RangeShifter model for pine martens reintroduced to England and Wales, which shows reasonable fit to the observed data. We used this model to explore the spread of individuals from existing populations in southwest Britain, demonstrating population persistence, although not coalescence. We also found that realistic woodland scenarios do not improve pine marten dispersal or abundances in a variety of landscapes.

### 4.1 Calibration

Although we find calibration does improve some of our IBM parameter estimates, such as emigration probability, there are minimal differences between the priors and posteriors for most of the calibrated parameters. This is likely because we calibrated our model using binary presence/absence data, which contains less information than the abundance data used in the original method (Malchow et al. 2024). As monitoring of pine martens continues in Britain, through new projects (e.g., https://pinemartens.uk/national‐pine‐marten‐monitoring‐programme) and as part of grey squirrel policy in England (https://www.gov.uk/government/publications/grey‐squirrel‐policy‐statement/grey‐squirrel‐policy‐statement‐managing‐the‐impact‐of‐grey‐squirrels), additional data could be used to further calibrate this model.

Our calibrated model has good fit to all but one spatial fold. Spatial fold one, with the lowest AUC and sensitivity, encompassed North Wales and so the furthest observations from the known release sites. A small number of individuals travelled up to 100 km in the dispersal phase after release (McNicol et al. 2020) and it is possible that our model struggled to account for these unusual long‐distance movements through suitable habitat patches. Given that hyperdispersal is a common behaviour exhibited by translocated individuals (Bilby and Moseby 2024), our model may not be able to accurately reflect reintroduction scenarios, underestimating the spread of individuals and overestimating abundance close to the release areas. This may limit the accuracy of the simulated reintroductions when considering population spread in southwest Britain.

### 4.2 Spread in the Southwest

Our calibrated IBM shows strong population growth and persistence of pine marten populations in Southwest Britain, even though they may not form a contiguous population. Although the reintroductions to Wales and the Forest of Dean are likely to form a single population, those in the New Forest and further Southwest are likely to remain isolated. Although all of the reintroductions are designed to be self‐sustaining (MacPherson et al. 2014; Stringer et al. 2018), the isolation of some populations even after 150 years could have genetic consequences.

It was recently found that recovering pine marten populations in Scotland, where the founders of the reintroduced populations are sourced, have a very low effective population size (O'Reilly et al. 2025). Low effective population sizes are associated with poor long‐term viability and reduced evolutionary potential. As such, the reintroduced population may also have similar low genetic diversity, which could be compounded by founder effects. In the long term, this means the reintroduced population could become less resilient and potentially experience decreases in survival and/or fecundity rates, reducing its long‐term viability. It will be important to maintain large populations of pine martens to mitigate the effects of low effective population sizes (O'Reilly et al. 2025), which might require further translocations. It could also be beneficial to source individuals from other genetic stock, such as from Europe which is genetically different from the Scottish population (Kyle et al. 2003). However, there are risks that European pine martens may not be behaviourally adapted to conditions in Britain (MacPherson and Wright 2021) and may introduce new diseases to the British island population. Our model can be adapted to aid in identifying release locations which would maximise the chance of merging populations and could be expanded to account for genetic effects (Malchow et al. 2021).

Our models suggest that the Devon and Somerset reintroduction may result in small, isolated populations after 150 years with a possible loss of the population released in Exmoor (Somerset). This differs from other studies (MacPherson et al. 2024) and the initial feasibility report for the proposed reintroduction (MacPherson et al. 2021) whose population viability analyses suggest the establishment of a small but stable long‐term population. This difference could be due to our conservative definition of suitable habitat patches and the lack of future habitat change included in the projection, which are explored in the limitations section.

### 4.3 Woodland Creation Scenarios

**FIGURE 9:**
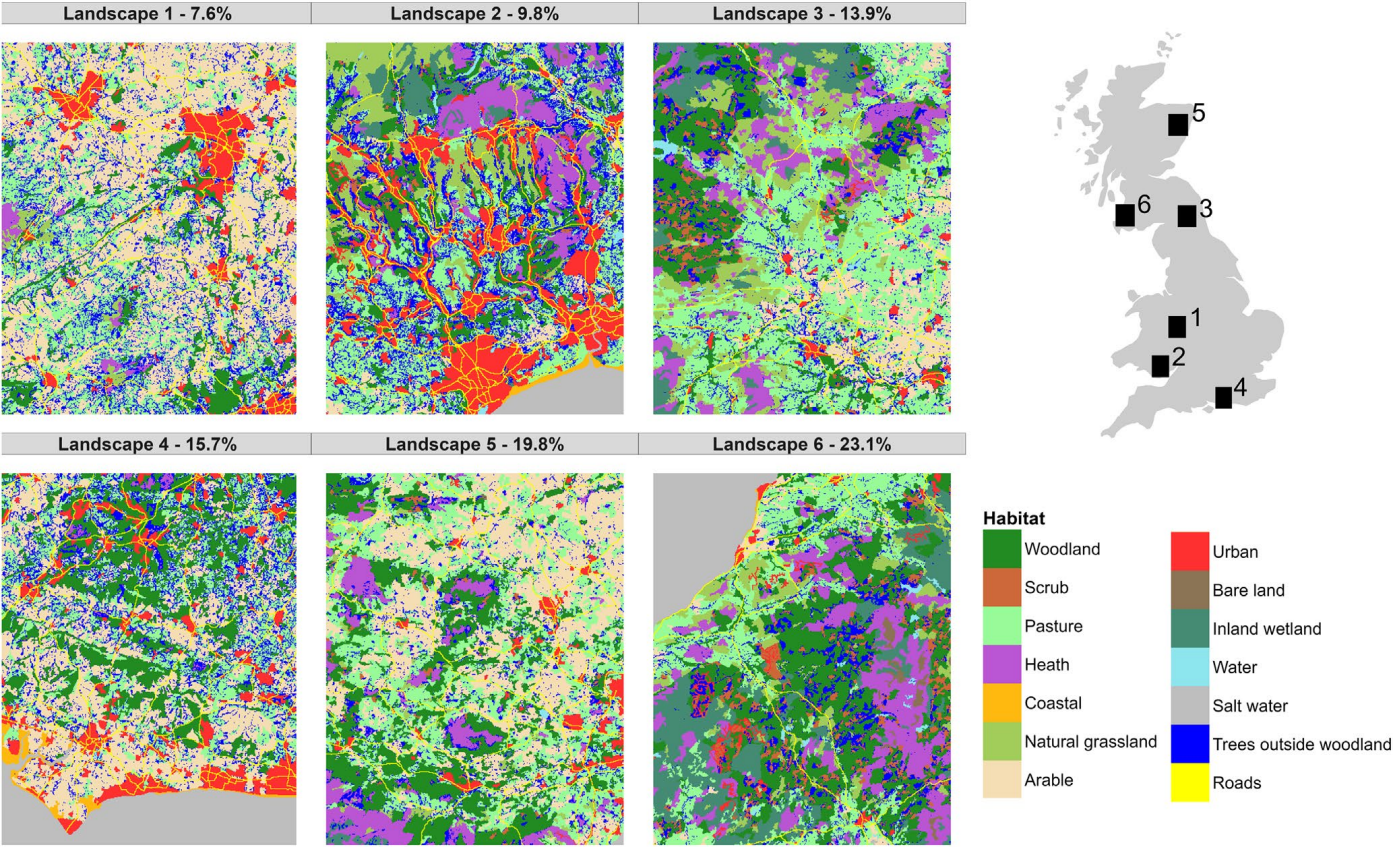
The six randomly selected landscapes for testing woodland creation scenarios. The inset map (top right) shows the location of each landscape sample within Britain. The landscape name and the proportion of woodland (%) is shown in each plot title. The map colours show the 14 habitat categories. Each landscape is 40 km × 50 km.

Our scenario analysis demonstrates that, with sufficient woodland coverage, reintroduced populations can establish quickly. There is a marked difference between Landscape 2 and 3 (Figure 8) in the establishment of a pine marten population, despite only 4% (or 80 km^2^) difference in woodland cover. Potentially, this increase in woodland cover provided sufficient resident habitat for the population to establish and disperse more effectively. However, the selected landscapes differ in more than just the proportion of woodland cover. Landscape 2 has much greater proportions of urban habitat, and the woodlands appear smaller and more fragmented compared to landscape 3 (Figure 9). The differences in the configuration of woodland, and the composition of the intervening matrix, between the six selected landscapes are a limitation of this study. We used randomly selected British landscapes to reflect realistic configurations of the 14 habitat types, which would be difficult to simulate. However, this means we could not control for patch size and distribution whilst only altering the proportion of woodland cover, which makes it difficult to compare results across landscapes. Landscape has the greatest impact on abundance, but it is not possible to ascertain whether this is driven by proportion of woodland cover or other differences.

Focusing on comparing within the landscapes, we find that existing levels of woodland creation, regardless of the configuration, are unlikely to have impacts on pine marten abundance and distribution at our tested scale. We chose to explore how realistic areas of woodland creation might impact pine marten populations. However, this means that most of the simulated creation areas are smaller than our minimum suitable patch size (2 km^2^, Figure S1). This means no new patches are created during woodland planting and the carrying capacity of the landscape remains the same. This suggests that existing schemes may not encourage planting at sufficient scale to meaningfully increase pine marten populations. Instead, the proportion of woodland in the landscape would have to markedly increase to create new areas of at least 2 km^2^, which may not be possible given competing land use goals. However, this level of change is occasionally possible through the creation of National Forests and via landscape recovery schemes emphasising woodland creation. The existing National Forest increased woodland cover from 6% in 1991 to 25% in 2023 (https://www.nationalforest.org/about/our‐history) and the Environmental Improvement Plan (2025; https://www.gov.uk/government/publications/environmental‐improvement‐plan‐2025/environmental‐improvement‐plan‐eip‐2025) lists a commitment to create three new National Forests. The reliance on existing woodlands suggests that there needs to be greater emphasis on maintaining the quality and resilience of these areas rather than just focussing on creation of new woodlands.

### 4.4 Limitations

Our definition of suitable habitat patches means there are few suitable patches in the area that connects some of the reintroductions, such as between Somerset and Devon (Figure 4), and means that realistic woodland creation does not add new patches of residential habitat to the landscape. This definition of suitable patches is conservative but is based on habitat suitability analyses (MacPherson and Wright 2021; Scopes et al. 2025) and published studies of minimum requirements for pine marten territory establishment (Zalewski and Jedrzejewski 2006). Further, other population viability studies for British pine martens have used 2 km^2^ of very suitable habitat as the minimum home range of an individual (MacPherson and Wright 2021).

Pine martens can persist in a network of smaller woodlands connected by foraging habitat or TOW (Mergey et al. 2023), however this is difficult to model with the chosen software. Establishing territories in such habitat networks could enable population persistence in wider areas of Britain where large woodlands are absent, perhaps allowing population coalescence in southwest Britain. It may also mean that we have underestimated the impact of woodland creation, as our current model cannot account for instances where the creation of a small woodland establishes a viable habitat network. Future work could consider using habitat suitability outputs directly as the landscape instead of a patch‐based model, which was not feasible with our methods due to the focus on woodland creation.

Furthermore, pine martens use hedges and small patches of woodland to disperse and within daily movements (e.g., for foraging; Manzo et al. 2018), which may therefore reduce the mortality risk associated with open habitats and roads. Such small‐scale habitats are likely to be underestimated in our model, given its spatial resolution. We used a coarse resolution to calibrate against a large area of Southwest Britain, but a higher resolution model could reveal greater use of linear features, which have limited representation in 1 ha pixels. Small‐scale woodland creation may therefore impact survival in a way which has not been included in our model. However, it is also clear that individuals can move large distances across mixed landscapes (McNicol et al. 2020) suggesting that small scale planting may not impact dispersal capability but rather the paths chosen.

Future monitoring could refine our understanding of how pine martens use small patches and linear features, both for dispersal and possibly as part of their territory. This would make use of higher resolution telemetry data, which is becoming more feasible as GPS units reduce in size (Stevenson et al. 2013), and higher resolution data on TOW, which has become available since analysis (Forest Research, https://environment.data.gov.uk/dataset/9c41b3c6‐2453‐44f6‐9900‐e7821f1a1072).

One further limitation of this study is that the landscape was static for the simulated 150 years as we cannot predict land use changes, which could have significant impacts on the population distribution. Reaching current government environmental targets, such as the creation of 500,000 ha of wildlife‐rich habitat by 2042 (Environment Act, 2021), would likely increase the amount of habitat available for pine martens in the Southwest and may have further improved the outcomes of our simulation if they had been included. We also only considered a single addition of woodland, rather than dynamic changes through time, which may underestimate the impact of woodland creation on pine marten abundance.

### 4.5 Summary

Our calibrated individual‐based model suggests that, following multiple reintroductions, pine martens are likely to persist in England and Wales into the near future. The model likely underestimates connectivity between the existing populations due to our conservative definition of habitat patches and coarse scale of modelling, but still demonstrates some movement between reintroduction schemes. Our model therefore demonstrates the success of the reintroduction programme and long‐term strategy for pine marten conservation in Britain (MacPherson and Wright 2021). Further, our model suggests that current levels of woodland creation are unlikely to impact pine marten abundance or dispersal at the modelled scale. However, more spatially detailed analysis is needed to investigate how small‐scale woodland creation might impact pine marten habitat selection and dispersal paths.

## Author Contributions

**Eleanor R. Scopes:** conceptualization (equal), data curation (equal), formal analysis (lead), writing – original draft (lead). **Nora Kerecsenyi:** data curation (equal), writing – review and editing (equal). **Kevin Watts:** funding acquisition (equal), writing – review and editing (equal). **Jenny MacPherson:** conceptualization (equal), supervision (equal), writing – review and editing (equal). **Patrick G. R. Wright:** conceptualization (equal), supervision (equal), writing – review and editing (equal). **Catherine M. McNicol:** conceptualization (equal), supervision (equal), writing – review and editing (equal). **Jamie Kingscott‐Edmunds:** conceptualization (equal), data curation (equal), writing – review and editing (equal). **Cally Ham:** conceptualization (equal), writing – review and editing (equal). **Matt Guy:** conceptualization (equal), writing – review and editing (equal). **Chloe C. Bellamy:** conceptualization (equal), supervision (equal), writing – review and editing (equal).

## Funding

This work was supported by the Department for Environment, Food and Rural Affairs, UK Government through Defra's Nature for Climate Fund program.

## Disclosure

Statement of inclusion: Our study was located primarily in the UK and all our authors come from this country. This study is part of a collaboration between Forest Research, Vincent Wildlife Trust and Gloucestershire Wildlife Trust, and the authors list has representatives from each institution.

## Conflicts of Interest

The authors declare no conflicts of interest.

## Supporting information


**Table S1:** The simplified habitat groupings used in the RangeShifter model of pine marten life history dispersal. The table shows the group names and the names and numbers of the Corine 2018 land cover classifications (Büttner et al. 2004) included in that group.
**Table S2:** The RangeShifter parameter values and settings. This is a stage‐structured simple sexual model with overlapping generations. The table indicates the values which are calibrated by Bayesian inference, and are specified using priors and posteriors. References: (a) Ruette et al. (2015); (b) Powell et al. (2012); (c) Stringer et al. (2018); (d) Amstislavsky and Ternovskaya (2000); (e) Zalewski and Jedrzejewski (2006); (f) Harris and Yalden (2008); (g) Malchow et al. (2024); (h) Scopes et al. in prep.; (i) Ovenden et al. (2019).
**Table S3:** Habitats and their associated cost value for the stochastic movement model and their per‐step mortality. Costs and mortality probabilities are relative to woodland, which is the primary habitat for pine martens (MacPherson and Wright 2021). Each step is 100 m.
**Figure S1:** An example scenario map in Landscapes one and six, where woodland patches have been randomly added to the map, and the areas are drawn from the largest 50% of the England Woodland Creation Offer data. The suitable habitat patches, based around woodland > 2 km^2^ are shown in light blue, and the newly created patches under the scenario in red. The other habitats are shown in greyscale to aid visualisation, but can but viewed in Figure 6. Each landscape is 40 km × 50 km.

## Data Availability

The code for the calibrated model, investigation of the spread of pine martens in Southwest Britain and woodland creation scenarios is available at Zenodo repository (https://doi.org/10.5281/zenodo.20283313). Model calibration code will not be provided as the field data used in this process is sensitive.
