# Supplementary material for: Individual‐Based Models Reveal Population Recovery and Impacts of Woodland Creation on Pine Martens ( Martes martes ) in Britain
Source: Ecol Evol. 2026 Aug 13;16(8):e74175. doi: 10.1002/ece3.74175 (PMC13473748; doi:10.1002/ece3.74175)
Supplement: Supplementary file 1 [file ECE3-16-e74175-s001.docx]

**Supporting Information**

***Table S1. The simplified habitat groupings used in the RangeShifter model of pine marten life history dispersal.*** *The table shows the group names and the names and numbers of the Corine 2018 land cover classifications (Büttner et al., 2004) included in that group.*

| **Group** | **Corine category name** | **Corine number** |
| --- | --- | --- |
| Woodland | Broad-leaved forest; Coniferous forest; Mixed forest | 3.1.1, 3.1.2, 3.1.3 |
| Scrub | Transitional woodland-shrub | 3.2.4 |
| Pasture | Pastures | 2.3.1 |
| Heath | Moors and heathland | 3.2.2 |
| Coastal | Salt marshes; Intertidal flats; Beaches, dunes, sands | 4.2.1., 4.2.3, 3.3.1 |
| Natural grasslands | Natural grasslands | 3.2.1 |
| Arable | Non-irrigated arable land; Fruit trees and berry plantations; Complex cultivation patterns; Land principally occupied by agriculture, with significant areas of natural vegetation; Agro-forestry areas | 2.1.1, 2.2.2, 2.4.2, 2.4.3,  2.4.4 |
| Urban | Continuous urban fabric; Discontinuous urban fabric; Industrial or commercial units; Road and rail networks and associated land; Port areas; Airports; Mineral extraction sites; Dump sites; Construction sites; Green urban areas; Sport and leisure facilities | 1.1.1, 1.1.2, 1.2.1, 1.2.2,  1.2.3, 1.2.4, 1.3.1, 1.3.2,  1.3.3, 1.4.1, 1.4.2 |
| Bare land | Bare rocks; Sparsely vegetated area; Burnt areas | 3.3.2, 3.3.3, 3.3.4 |
| Inland wetlands | Inland marshes; Peat bogs | 4.1.1, 4.1.2 |
| Water | Water courses; Water bodies | 5.1.1, 5.1.2 |
| Salt water | Sea and ocean; Coastal lagoons; Estuaries | 5.2.3, 5.2.1, 5.2.2 |

***Table S2. The RangeShifter parameter values and settings.*** *This is a stage-structured simple sexual model with overlapping generations. The table indicates the values which are calibrated by Bayesian inference, and are specified using priors and posteriors. References: (a) Ruette et al. 2015; (b) Powell et al. 2012; (c) Stringer et al. 2018; (d) Amstislavsky and Ternovskaya 2000; (e) Zalewski and Jedrzejewski 2006; (f) Harris and Yalden 2008; (g) Malchow et al. 2024; (h) Scopes et al. in prep.; (i) Ovenden et al. 2019.*

| **Demographic parameters** | **Value** |
| --- | --- |
| Number of stages^(a, b)^ | 3 |
| Juvenile survival probability | Calibrated |
| Sub-adult survival probability | Calibrated |
| Adult survival probability | Calibrated |
| Fecundity | Calibrated |
| Maximum age (years)^(c)^ | 15 |
| Number of reproduction seasons per year^(d)^ | 1 |
| Survival density dependence | Calibrated |
| Probability of being male at birth^(e)^ | 50% |
| Probability of reproducing annually | 100% |
| Number of years after reproduction before subsequent reproduction^(f)^ | 0 |
| **Dispersal parameters** | **Value** |
| Sub-adult emigration probability | Calibrated |
| Maximum settlement probability^(g)^ | 1 |
| Slope of density-dependence on settlement^(g)^ | -5 |
| Settlement slope beta - inflection point of density-dependence relationship | Calibrated |
| Maximum number of steps per year^(h)^ | 700 |
| Maximum number of steps in lifetime | 5000 |
| **Stochastic movement simulator values**  Perceptual range^(i)^ | 5 |
| Perceptual range method^(i)^ | 2 (harmonic mean) |
| Memory size (steps)^(i)^ | 5 |
| Directional persistence^(i)^ | Calibrated |
| Goal type^(i)^ | 0 |

***Table S3. Habitats and their associated cost value for the stochastic movement model and their per-step mortality.*** *Costs and mortality probabilities are relative to woodland, which is the primary habitat for pine martens (MacPherson and Wright, 2021). Each step is 100m.*

| **Habitat** | **Cost value** | **Per-step probability** | **mortality** |
| --- | --- | --- | --- |
| Woodland | 1 | 0 |  |
| Scrub | 1 | 0.00001 |  |
| Pasture | 20 | 0.0002 |  |
| Heath | 10 | 0.00001 |  |
| Coastal | 50 | 0.0002 |  |
| Natural grasslands | 15 | 0.0001 |  |
| Arable | 30 | 0.0002 |  |
| Urban | 100 | 0.0005 |  |
| Bare land | 100 | 0.0003 |  |
| Inland wetlands | 100 | 0.001 |  |
| Water | 100 | 0.001 |  |
| Salt water | 100000 | 0.99 |  |
| Trees outside woodland | 1 | 0.00001 |  |
| Roads | 100 | 0.07 |  |

***Fig. S1. An example scenario map in Landscapes one and six, where woodland patches have been randomly added to the map, and the areas are drawn from the largest 50% of the England Woodland Creation Offer data.*** *The suitable habitat patches, based around woodland >2km^2^ are shown in light blue, and the newly created patches under the scenario in red. The other habitats are shown in greyscale to aid visualisation, but can but viewed in Figure 6. Each landscape is 40 km X 50 km.*

**References**

Amstislavsky, S. and Ternovskaya, Y. (2000), ‘Reproduction in mustelids’, *Animal Reproduction Science* **60**, 571–581.

Büttner, G., Feranec, J., Jaffrain, G., Mari, L., Maucha, G. and Soukup, T. (2004), ‘The CORINE land cover 2000 project’, *EARSeL eProceedings* **3**(3), 331–346.

Harris, S. and Yalden, D. W., eds (2008), *Mammals of the British Isle: Handbook*, 4 edn, The Mammal Society, Southampton.

MacPherson, J. and Wright, P. (2021), Long-term strategic recovery plan for pine martens in Britain, Technical report, Vincent Wildlife Trust, Ledbury, UK.

Malchow, A., Fandos, G., Kormann, U. G., Grüebler, M. U., Kéry, M., Hartig, F. and Zurell, D. (2024), ‘Fitting individual-based models of spatial population dynamics to long-term monitoring data’, *Ecological Applications* p. e2966.

Ovenden, T. S., Palmer, S. C., Travis, J. M. and Healey, J. R. (2019), ‘Improving reintroduction success in large carnivores through individual-based modelling: How to reintroduce Eurasian lynx (Lynx lynx) to Scotland’, *Biological Conservation* **234**, 140–153.

Powell, R. A., C, J., Lewis, Slough, B. G., Brainerd, S. M., Jordan, N. R., Abramov, A. V., Monakhov, V., Zollner, P. A. and Murakami, T. (2012), Evaluating translocations of martens, sables, and fishers: testing model predictions with field data, *in* K. B. Aubry, W. J. Zielinski, M. G. Raphael, G. Proulx and S. W. Buskirk, eds, ‘Biology and Conservation of Martens, Sables and Fishers: A New Synthesis’, Cornell University Press, USA, pp. 93–137.

Ruette, S., Vandel, J., Albaret, M. and Devillard, S. (2015), ‘Comparative survival pattern of the syntopic pine andstone martens in a trapped rural area in France’, *Journal of Zoology* **295**(3), 214–222.

Scopes, E., MacPherson, J., Wright, P., McNicol, C., Kingscott-Edmunds, J., Ham, C., Kerecsenyi, N. and Guy, M. a. B. C. (in prep.), ‘Validating habitat suitability models for pine marten (*Martes martes*) reintroductions to England and Wales’.

Stringer, A., MacPherson, J., Carter, S., Gill, R., Ambrose-Oji, B., Wilson, R., Kelsall, P., Feirn, W., Galbraith, L., Hilário, C., Parry, G. and Taylor, A. (2018), The feasibility of reintroducing pine martens (*Martes martes*) to the Forest of Dean and lower Wye Valley, Technical report, Woodland Trust, Grantham, UK.

Zalewski, A. and Jedrzejewski, W. (2006), ‘Spatial organisation and dynamics of the pine marten Martes martes population in Bial owiez˙a Forest (E Poland) compared with other European woodlands’, *Ecography* **29**(1), 31–43.
